# Culturally adapted motivational interviewing with cognitive behavior therapy and mindfulness-based relapse prevention for substance use disorder in Pakistan (CAMAIB): protocol for a feasibility factorial randomised controlled trial

**DOI:** 10.1186/s40814-023-01296-0

**Published:** 2023-04-24

**Authors:** Muqaddas Asif, Ameer B. Khoso, M. Ali Husain, Salman Shahzad, Marie-Claire Van Hout, Noor-ul-Zaman Rafiq, Steven Lane, Imran Bashir Chaudhry, Nusrat Husain

**Affiliations:** 1 Pakistan Institute of Living and Learning, Karachi, Pakistan; 2 St. Helens and Knowsley NHS Trust, Prescot, UK; 3 University of Karachi, Karachi, Pakistan; 4 Liverpool John Moores University, Liverpool, UK; 5 Phoenix Foundation for Research and Development, Lahore, Pakistan; 6 The University of Liverpool, Liverpool, UK; 7 Ziauddin University Hospital, Karachi, Pakistan; 8 The University of Manchester, Manchester, UK

**Keywords:** Cognitive behavioral therapy, Motivational interviewing, Mindfulness-based relapse prevention, Substance use disorder, Pakistan

## Abstract

**Background:**

The use of psychoactive substances significantly impacts the health, social and economic aspects of families, communities and nations. There is a need to develop and test psychological interventions aimed for individuals with substance use disorder (SUD) in lower- and middle-income countries (LMICs), such as in Pakistan. The aim of this exploratory trial is to test the feasibility and acceptability of two culturally adapted psychological interventions in a factorial randomised controlled trial (RCT).

**Methods:**

The proposed project will be conducted in three phases. The first phase of the study will focus on cultural adaptation of the interventions through qualitative interviews with key stakeholders. The second phase will be to refine and produce manually assisted interventions. Third and last stage would be to assess the feasibility of the culturally adapted interventions through a factorial RCT. The study will be carried out in Karachi, Hyderabad, Peshawar, Lahore and Rawalpindi, Pakistan. Recruitment of participants will take place from primary care and volunteer organisations/drug rehabilitation centres. A total of 260 individuals diagnosed with SUD (*n* = 65) in each of the four arms will be recruited. The intervention will be delivered weekly over a period of 12 weeks in both individual and group settings. Assessments will be carried out at baseline, at 12th week (after completion of intervention) and 24th week post-randomisation. The analysis will determine the feasibility of recruitment, randomisation, retention and intervention delivery. Acceptability of intervention will be determined in terms of adherence to intervention, i.e. the mean number of sessions attended, number of home assignments completed, attrition rates, as well as through process evaluation to understand the implementation process, context, participants’ satisfaction, and impact of the study intervention. The health resource use and impact on the quality of life will be established through health economic data.

**Discussion:**

This study will provide evidence for feasibility and acceptability of culturally adapted manually assisted psychological interventions for individuals with SUD in the context of Pakistan. The study will have clinical implications if intervention is proven feasible and acceptable.

**Trial registration:**

Name of the registry: ClinicalTrials.gov, Trial registration number: NCT04885569, Date of registration: 25th April 2021.

**Supplementary Information:**

The online version contains supplementary material available at 10.1186/s40814-023-01296-0.

## Background

The use of psychoactive substances significantly impacts the health, social and economic aspects of families, communities and nations. In 2019, approximately 275 million people worldwide used illicit substances with almost half a million deaths related to substance misuse [1]. Substance misuse has resulted in 18 million years of healthy life lost [1]. Substance misuse has increased 22 percent in last 10 years and it is thought that the number of people using illicit substance will increase further 11% by 2030 [1]. According to the World Drug Report [2], only 1 out of 8 people receive the required treatment for substance misuse. In Pakistan, 7.6 million people use illicit substances and approximately 4.25 million people are dependent [3] with 50,000 more people becoming dependent on illicit substances each year [4]. This calls for urgent attention and treatment approaches for individuals with SUD [3]. In Pakistan, there is a higher prevalence of substance misuse amongst men compared to women [5]. Amongst men, cannabis and opioids have been the most used substances [5]. Heroin is consumed more in Pakistan than in any other country in the South Asian region [5]. In women, there is a higher prevalence of prescription opioids, tranquilizers and sedatives misuse [3].

Previous history of substance use, lack of family support and stress have been reported as underpinned associations of relapse [6]. Though there are strong religious and cultural objections to substance use, it is a highly prevalent problem amongst the youth of Pakistan. These findings suggest an increased burden on public health resources. The majority of people who use illicit substances belong to the poor strata of the society [3]. With most detoxification centres in Pakistan being operated by non-governmental organizations (NGOs), access to treatment is limited. Around 75% of people dependent on opiates are left without an avenue to seek help (Bureau of International Narcotics and Law Enforcement Affairs [7]. It is therefore necessary to address needs of this population in order to provide effective and targeted interventions.

Evidence suggests that psychological interventions such as Cognitive Behavioural Therapy (CBT), Motivational Interviewing (MI) and Relapse Prevention (RP) interventions are effective treatments for substance misuse [8]. CBT is considered as the most effective psychological treatment for SUD [9, 10]. The CBT approach comprises of different types of interventions [11] such as CBT based MI [12], mindfulness based stress reduction [13], Mindfulness-Based Relapse Prevention (MBRP) [14] which can either be combined or used as standalone interventions. Although, CBT focuses on unpleasant or distressing events, thoughts and actions, different approaches of CBT may aim to reduce the impact of internal triggers and promote coping skills to maintain abstinence in the long term [15]. For example, CBT-based MI (MICBT) for substance misuse focus on improving motivation to change (to improve access and engagement with the services) and on prevention of relapse. MI is effective in improving motivation to change [16] and to produce significant change in multiple substance related outcomes [17–19]. The addition of MI assessment has been shown to increase engagement and adherence to overall treatment [20].

In addition to MICBT, RP is another commonly used CBT intervention for SUDs developed to help clients maintain abstinence by identifying internal and external triggers and promoting effective coping skills that increase self-efficacy in managing triggers and reduce the risk of relapse [20]. Furthermore, MBRP is an extension of CBT-based RP approach that focuses on identifying triggers, thoughts and cravings, automatic reactions to those triggers, and using mindfulness as a way of effectively coping with situations that may trigger relapse [21, 22]. Mindfulness-based interventions have been increasingly developed and evaluated for the treatment of SUD [23]. Although, CBT and its different approaches, i.e., MICBT and MBRP have shown to be effective interventions for substance misuse, these are widely used in high-income countries [24] and there is high degree of heterogeneity as reported in a previous systematic review [25]. So, the evidence of the efficacy of MICBT and MBRP for SUD in LMICs is scarce. There is also an identified need to culturally adapt CBT for LMICs [10]. In Pakistan, there has been very little research in this area.

## Study aims and objectives

The study aims to culturally adapt and integrate two CBT approaches, MICBT and MBRP intervention for individuals with SUDs in Pakistan and to test the feasibility and acceptability of these interventions in a factorial RCT.

## Methods

### Study design and setting

**Fig. 1:**
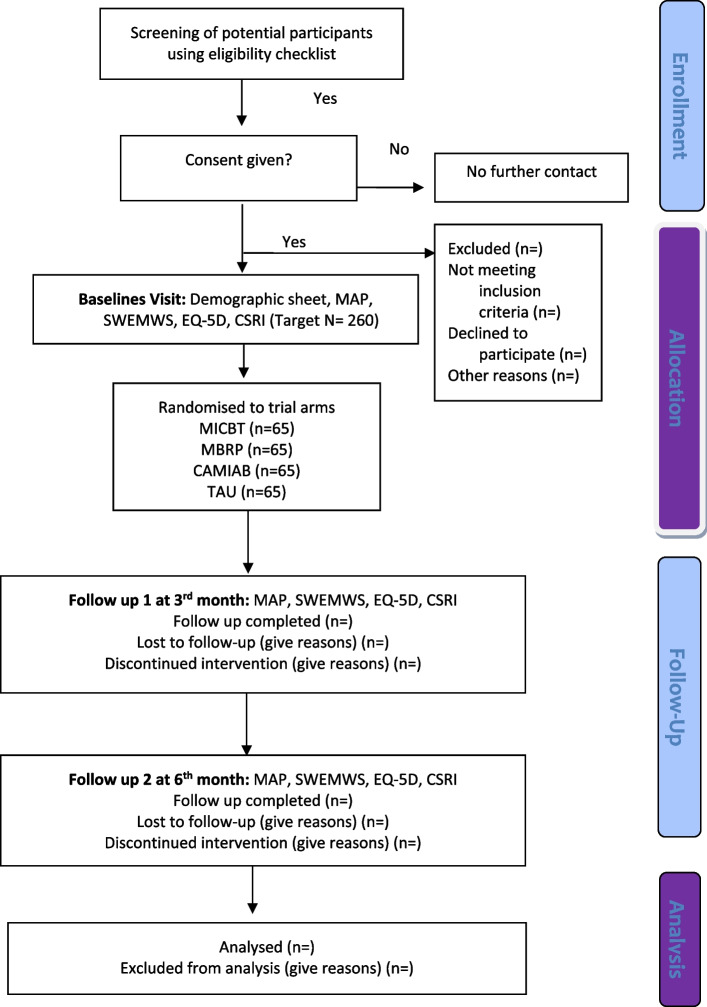
Flow diagram describing the timeline of study procedures

This will be a four-arm feasibility factorial RCT. An overview of complete study design is provided in Fig. 1. The proposed project will consist of three phases. The first phase of the study will focus on the adaptation/development of the intervention in accordance with the Pakistani culture. The second phase will include refinement of the interventions and production of the intervention manuals. The third phase will be to assess the feasibility through a factorial RCT. The study will be carried out in Karachi, Hyderabad, Peshawar, Lahore and Rawalpindi, Pakistan. Recruitment of participants will be from primary care and volunteer organisations/drug rehabilitation centres.

### Phase 1: intervention adaptation/development

The first stage of the study will comprise of focus groups and in-depth interviews with service users and other stakeholders. The purpose of this phase is the cultural adaptation of the evidence-based MICBT [12, 26, 27] and MBRP [28, 29] for the study population. Through qualitative work, the culturally adapted intervention will be developed for people with SUD, as well as an accompanying manual. Focus groups will be conducted with key stakeholder groups including (1) service users, (2) family members/carers, (3) health professionals and (4) Community leaders, religious scholars (Imams) (8–10 in each group; total sample *n* = 32–40). We will also conduct 1:1 individual interviews if participant is not willing for focus group discussion.

There will be active selection of participants in order to represent the wider population. Focus group interviews will be conducted by a trained research assistant, lasting between 60 and 90 min—including the time for setting up and addressing participants’ queries. The topic guide will be developed in collaboration with the members from the service user and carer’s advisory group. For consistency, the same topic guide will be used with participants who prefer individual interviews. Data will be analysed using thematic analysis [30] for emergent themes relevant to the cultural adaptation of MICBT and MBRP. The following areas will be considered specifically (1) experiences with the services, (2) opinions on the cultural adaptation of MICBT and MBRP intervention, (3) identifying and understanding barriers limiting access to services, and (4) opinions on how best to involve family in the treatment plan. Our group has culturally adapted interventions for a range of mental disorders using mixed methods in Pakistan and the UK and we will follow same procedures described elsewhere [31–39].

### Phase 2: test run and intervention refinement

Phase 2 will involve preliminary testing of the culturally adapted intervention in a small group of participants. Cultural adaptation of the intervention requires an understanding of the religious and socio-cultural constructs of the population. This will take into consideration the explanatory models of any illness, along with understanding of underlying beliefs and values [31]. Such adaptations are reported to increase the acceptability and uptake of psychological and social interventions. Once the cultural adaptation is complete, a sample of 12 participants diagnosed with SUD will be invited to participate in the test run of the intervention. This will consist of 8 individual and 4 group sessions focusing on distinct yet related stages: exploring motivation to change; exploring the process of change; identifying strategies to consolidate commitment to change, explore effective coping skills, increase awareness and development of flexible responding in the presence of triggers of relapse. Feedback will be gathered from the participants, therapists and staff, on the relevance and acceptability of the culturally adapted interventions. All participants of trial run will be invited to be interviewed to explore their opinions on intervention relevance and acceptability. All interviews will be carried out by researchers from outside the local community in order to further protect interviewee confidentiality. These interviews will be recorded and transcribed. The interview guide will include some predetermined questions (based on findings from phase 1) and some open-ended questions to facilitate emergence of new themes.

### Phase 3: feasibility factorial RCT

The feasibility of the intervention will be assessed using a factorial RCT, for MICBT and MBRP intervention, comparing four groups: (1) MICBT plus treatment as usual group (TAU), (2) MBRP plus TAU group, (3) the integrated MICBT and MBRP with TAU and (4) TAU group alone. In Pakistani culture, people consider themselves more as part of a family and larger community rather than as individuals. Therefore, a group approach is more culturally appropriate but for certain private/family issues the flexibility of some individual time with the therapists will be offered. The group sessions will consist of approximately eight participants.

### Eligibility criteria

Participants meeting DSM-V criteria for SUD, aged 18 years and above, understand spoken Urdu and have completed the detoxification process (to ensure engagement in the intervention) will be included. Exclusions will be made if there will be any evidence of organic brain disease, clinically significant co-occurring medical illness or mental illness to the extent that can prevent participation in the intervention. A trained research assistant (RA) will confirm participants’ eligibility against inclusion/exclusion criteria and obtain informed consent.

### Interventions

#### Explanation for the choice of comparators

MI is being increasingly blended with treatments such as CBT to enhance intrinsic motivation for behavioural change and found to have positive outcomes [40]; however, further research is required to replicate these findings in LMICs such as Pakistan. Similarly, a recent review of 54 RCTs supports efficacy of mindfulness-based interventions for SUDs and RP [29]. However, the review also calls for more rigorous research designs with longer follow-up periods [29]. The review concluded that the most effective intervention approach to target SUD is to combine mindfulness interventions with treatment-as-usual (TAU) or other active treatments. Another review by Garland and Howard [41] has also provided strong recommendations for the next wave of research to firmly establish the efficacy of mindfulness interventions for SUD.

### Intervention description

#### CBT-based Motivational Interviewing (MICBT)

The culturally adapted MICBT [26, 27] includes approaches of CBT based motivational interviewing. MI is a well-developed intervention for exploring and resolving the ambivalence to change behaviour. MICBT [27] includes techniques to increase and consolidate motivation to make positive changes in individuals with problematic substance use, cognitive restructuring, learning assertiveness and problem solving skills combined with cognitive behavioural relapse prevention approaches. The cognitive behavioural model of substance use suggests that problematic substance use is maintained by thoughts of reduced ability to cope when faced with high-risk situations. Beliefs regarding inability to cope with difficulties interact with physiological cravings and psychological urges to use. During this time, the decision to use or continue to use substances is made. Relief from cravings and urges occur and reinforce the belief that substance use is the only way to manage such situations. This intervention will focus on ambivalence to change substance use behaviour. This will also identify and help solve difficulties in changing substance use behaviour by offering ways of managing these difficulties through CBT.

#### Mindfulness based Relapse Prevention (MBRP)

MBRP [28] incorporates mindfulness-based practices with cognitive-behavioural skills to reduce likelihood of relapse, increase awareness and flexible responding towards triggers of SUD. Rationale of MBRP practices is its explicit emphasis to increase awareness to external triggers and discrete emotional, behavioural and cognitive reaction patterns are focused during initial sessions of intervention [28]. Exercises and practices explore the functions of habitual behaviours such as substance misuse and focus on the balance between acceptance of present moment and skilful action to bring real change. Moreover, MBRP put emphasis on association of thought content and relapse cycle and encouraged to focus on thoughts simply as thoughts and not essentially as reality. The latter sessions of MBRP centre on sustainability of learned skills in daily life, and to identifying or establish access to resources to strengthen supportive social networks [28].

#### CAMIAB intervention

This will be MICBT integrated with MBRP intervention. The interventions will be manualised and will include both individual as well as group sessions.

#### Treatment As Usual (TAU)

We will carefully record the routine care treatment that the participants will be receiving.

The intervention will be delivered alongside TAU in all study arms and participants will not be asked to cease any practices that they are currently undertaking on either control or intervention arm. Existing services received will be monitored and recorded throughout the trial. Intervention sessions will be delivered according to adapted intervention manuals. Manually assisted interventions help standardise the intervention in different settings and benefit in monitoring intervention fidelity.

### Criteria for discontinuing or modifying allocated interventions

The study interventions outlined are not expected to cause any adverse effects nor there is any anticipated harm to come from study participation. However, participants will be free to withdraw from the study at any point. The intervention will be discontinued if any individual participant requests to withdraw from the study or if any adverse effects warrant terminating the intervention. Given that this is a feasibility study, there will be no modifications in allocated treatments but a process evaluation will be undertaken to refine the intervention for a future definitive trial. After completion of intervention session, participants in need of further clinical interventions or who have not responded to intervention and those in TAU group will be referred to appropriate services.

### Strategies to improve adherence to interventions

Intervention sessions will be delivered according to adapted manuals and the intervention adherence will be monitored by number of sessions attended. The intervention will be delivered by trained therapist in supervision of senior therapist who have training and experience in conducting psychological interventions for SUD.

### Outcomes

#### Primary outcomes: feasibility indicators

Feasibility will be determined by following indicators:

- Recruitment rates, attendance (number of sessions attended), retention rate and randomisation.
- Acceptability of interventions based on client satisfaction, adherence rate (the number of sessions attended, number of home assignments completed) and attrition rates.
- Completeness of assessment by participants.

Meeting the progression criteria of > 50% of recruitment, > 70% of retention, > 60% of attendance, > 70% of adherence, < 30% of attrition and > 70% of assessment completion at study end will determine that a full definitive trial is needed.

#### Secondary outcomes

*The Maudsley Addiction Profile (MAP)* [42] is a brief questionnaire to measure problems in four domains that include substance use, health risk behaviour, physical and psychological health, and personal/social functions.

*The Short Warwick-Edinburgh Mental Well-being Scale (SWEMWBS)* [42] will be used to measure mental wellbeing. It consists of 7 statements on a 5-point Likert scale. The minimum scale score is 7 and the maximum is 35. The high score indicates better mental wellbeing.

*Quality of Life* [43] This will be measured using EuroQoL (EQ-5D) a standardised instrument to measure health outcomes and quality of life.

*Client Service Receipt Inventory (CSRI)* [44] will be used to estimate the health and social services received.

### Study procedure

The study procedure is outlined in Fig. 1 and in the Standard Protocol Items: Recommendations for Interventional Trials (SPIRIT) diagram (Table 1). A completed SPIRIT checklist is given as Additional file 1.

**Table 1:**
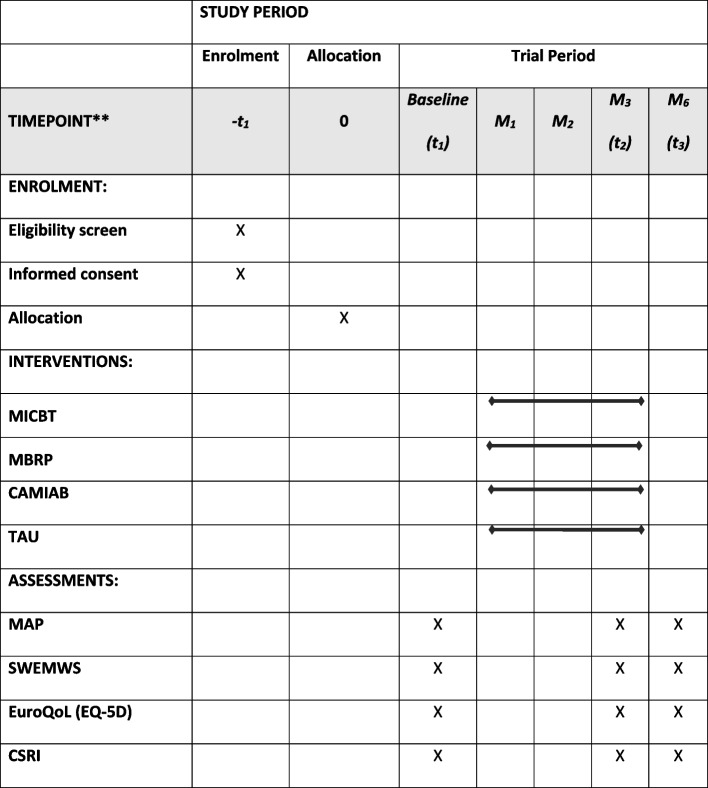
Standard Protocol Items: Recommendations for Interventional Trials (SPIRIT) diagram

Schedule of enrolment, interventions and assessments

#### Sample size

Based on the recommendations by Sim and Lewis [45], a minimum sample size of at least 50 participants per group is required for feasibility studies. Assuming 30% attrition rate, we aim to recruit a total of *N* = 260 participants, with 65 participants randomised to each arm according to 2 × 2 factorial design scheduled in given Table 2.

**Table 2:** 2 × 2 Factorial design schedule of intervention with sample size

	TAU	MBRP	Total
MICBT	65	65	130
TAU	65	65	130
Total	130	130	260

The findings of this feasibility study will help determine the initial data for the outcome measures and will help in providing a sample size estimate for a larger future definitive trial; therefore, no formal power calculations have been undertaken.

### Recruitment

Participants will be recruited from primary care and volunteer organisations/drug rehabilitation centres in Pakistan. A detailed Participant Information Sheet (PIS) about the study will be provided to all interested participants. Objectives as well as risks and benefits of participation will be detailed by a trained RA in local language. Participants eligible for study participation will be given at least 48 h to discuss with their family and to decide whether or not to take part. Those willing to participate will complete consent process and baseline assessments within 1 week of consent.

### Intervention allocation

#### Sequence generation

Participants will be randomly allocated to one of the four intervention arms by an independent offsite statistician using a computer-generated random sequence based on a block randomised design [46] until all study data are collected and verified. Once randomised, participants will be allocated a unique study identification number and informed about their intervention allocated groups within 1 week of randomisation.

#### Concealment mechanism

The independent statistician will share the randomised list of participants with the trial manager and he/she will further communicate the allocation information to therapists. It would not be possible to blind the staff delivering intervention and participants to treatment allocation but outcome assessments at each follow-up will be conducted by research staff blind to intervention groups. The therapist will advise participants not to discuss their intervention allocation with researchers doing outcome assessments.

#### Blinding

Those administering assessments at baseline and follow-ups, data entry, and data analysis will be blind to intervention groups. During the trial, periodic data quality checks will be carried out by the trial statistician. The trial statistician will be blinded to intervention allocations. Upon completion of data entry, preliminary data analysis will be carried out prior to un-blinding.

### Data collection method

All participants (intervention and TAU groups) will undergo assessments at baseline, 3rd month (end of intervention) and at 6th month after randomisation. Participants will complete a self- report demographic information sheet (only at baseline) that will collect information related to age, duration of substance misuse, type of substance used etc., the MAP [47], SWEMWBS [42], EuroQoL (EQ-5D) [43] and CSRI [44]. All the assessments will be carried out by trained research assistants at baseline, 3rd month (end of intervention) and at 6th month.

### Plans to promote participant retention and complete follow-up

Recruitment and retention are both an integral part of any drug and alcohol misuse trial due to the highly chaotic nature of this disorder. To increase retention of participants, protocols developed by Scott [48] will be used, where locater information will be recorded for each participant. Scott [48] achieved 95% retention of participants in his study on alcohol abuse. The study team will have regular contact with participants throughout the study period. Participants in the TAU group will be contacted by RA on weekly basis for first 3 months of the study to keep them engaged and retain in the study as well as to keep them well informed of the study’s progress. Both groups will be contacted monthly from 3 to 6 months to promote retention to the study. These contacts maybe done via a brief 5-min phone call.

### Data management

The data will be collected by trained RA’s to maintain confidentiality and anonymity. Only essential personal identifiable data will be collected in consent forms. Study data will be stored according to the General Data Protection Regulations 2018, with personal identifiable data secured separately. Electronic data will be stored in password protected computers. Demographic and questionnaire data will be anonymised immediately after collection and inputted into a study-specific password protected database. Paper copies of questionnaires and consent forms will be stored in locked cabinets. Personal identifiable data will be kept locked away, separate from other research data. Access to both hard copies and electronic versions of data will be restricted to only designated persons as approved by the site principal investigator (PI). A signed duty log will be used to access the data.

### Confidentiality

Participant’s confidentiality will be protected by assigning a unique study number and use of pseudonyms on all research material, e.g., transcripts. Reporting of verbatim quotations in presentations and publications will be carefully reviewed by the research team in advance. Paraphrasing will be used if there is any risk of identification of participants or third parties. The participants would be verbally informed of the possibility of confidentiality being broken in certain circumstances, prior to consent being sought. With participants’ consent, we will digitally record some of intervention session randomly to improve quality in clinical supervision. Recordings will be made on an encrypted digital voice recorder and uploaded to a password protected computer as soon as possible after completion. Recordings will be deleted from the recorder after uploading and destroyed in the presence of two researchers, after completion of analysis.

### Plans for collection, laboratory evaluation and storage of biological specimens for genetic or molecular analysis in this trial/future use

Not applicable.

### Data analysis

#### Statistical analysis

The study data will be analysed using SPSS (Version-22). Analysis of data will take place after full recruitment and data collection. Descriptive data analysis will be performed to summarise demographic data and feasibility outcomes. Baseline and outcome assessment data will be summarised using the appropriate descriptive statistics and graphical summaries. As this is a feasibility study, the analysis of the data will be largely descriptive and will focus on confidence intervals for the difference amongst groups for each outcome measure. Possible between-group differences will be assessed using analysis of variance (ANOVA) and chi-square tests. The results of the trial will follow the standard CONSORT recommendations. A complete statistical analysis plan will be written prior to database lock and subsequently will be discussed and approved by the Trial Steering Committee (TSC). There is no interim analysis planned.

#### Process evaluation

One to one semi-structured interviews will be conducted with randomly selected participants from each intervention group to explore the engagement with intervention, acceptability of the sessions, perceived benefits and barriers. We are anticipating 10–12 interviews from each intervention group to reach category saturation. We will also interview up to 2 therapists of each intervention group to explore their experiences of delivering the study interventions including the factors that can impact intervention such as any environmental or cultural factors. The interview will be conducted either face-to-face or through telephone. Interviews will last between 50 and 60 min and will be digitally audio recorded on an encrypted voice recording device. For analysis of interviews, thematic analysis [30] with constant comparison approach of qualitative evaluation will be used and major subthemes emerging from data will be presented. All interviews will be transcribed and paraphrased in Urdu language to capture the qualitative richness of the participants understanding. To maintain the credibility and trustworthiness of the data and subsequent findings, the researchers will be supervised by senior and experienced qualitative researchers. Engagement in discussion and regular reviews by senior researcher will ensure fit of the data to the final analysis, and helped to minimise bias [49]. Analysis and writing will be guided by a quality checklist [50].

#### Health economics

A preliminary assessment of the costs to implement study interventions (MICBT, MBRP and CAMIAB) will be conducted. A change in scores of EQ-5D from baseline to 24th weeks will also be conducted to determine feasibility of an economic evaluation for a future definitive trial.

#### Statistical methods to handle missing data

The groups will be analysed on an intention to treat basis. The analysis will determine effect sizes to conduct power calculations for a larger trial. In accordance with the intention to treat principles, our aim is to follow-up all participants regardless of whether they have been lost during the intervention. Any missing follow-up data will be modelled on the assumption that it is missing at random. Logistic regression will be used to test this assumption, and to calculate probability of providing complete outcome data with baseline variables as predictors. These analyses will be used to create inverse probability sampling weights for subsequent analyses on outcome.

### Ethical considerations

Ethical approval for the study has been obtained from National Bioethics Committee (NBC) of Pakistan (Ref: No. 4–87/NBC-467/20/47-Amend/21/1240). RAs will ask eligible participants to provide written informed consent for participation. Those participants who are not able to read and write will be provided with verbal information (in local language) that will encompass all the points mentioned in the written informed consent in presence of an independent trusted member who can read and write chosen by the participant. The consent form will then be thumb printed by the participants along with signatures of the legal representative chosen.

### Protocol amendments

Any amendments to the protocol will be communicated to NBC and other relevant authorities. The trial registration will be updated accordingly and revised protocol will be shared with research team and study investigators.

### Oversight and data monitoring

The research team will meet every month to monitor progress and decide on operative issues related to the project. The trial manager will be responsible to oversee co-ordination between study centres. An independently chaired TSC and Data Monitoring and Ethics Committee (DMEC) will oversee the trial throughout its various stages. This will be independent of the trial management team. TSC will meet every 6 months or with advice from the chair as needed between meetings. The DMEC will monitor the data and ethical aspects throughout the trial and advise the TSC on changes to the conduct of the study and whether there are any ethical or safety reasons why the trial should not continue. DMEC will consists of three independent members that collectively have expertise on mental health, statistics and health services research.

### Risk management: adverse event reporting and harms

Where a Serious Adverse Events (SAE), or Adverse Events (AE) occur for a particular participant, the research team will halt the study for that individual and review: (a) whether to withdraw the participant from the study, and (b) whether to halt the entire project. AEs will be recorded throughout the trial and any AE occurred will be immediately reported to PI within 24 h. This decision will be based on a consideration of the likelihood that the study itself (including the therapy and assessment procedures) contributed to the SAE (thus making it an Adverse Reaction; AR). Information will be gathered including the participant’s and researcher or therapist’s perspective, and the timing of the AE or SAE (e.g. did it occur immediately after a intervention session?) to help inform this review. Where an AR is identified, it will be considered whether this could apply to other participants and therefore if the study as a whole should be halted. This review process will be documented. Adverse Events (AE) are defined here as any untoward, time-limited, worsening in participants’ mental state or leading to a persistent increase in disability or incapacity. The occurrence of AEs will be monitored by the DMEC.

### Dissemination plans

Study findings will be published as widely as possible in peer reviewed and non-peer reviewed journals and will be presented at local participatory centres, and national and international conferences. Workshop and seminars will be organised in collaboration with established partnerships to disseminate research findings and also to increase awareness and reduce stigma associated with SUD. The study findings will also be disseminated through the PILL newsletter. We publish quarterly newsletters on various mental health problems which are circulated across Pakistan to primary care, secondary care, colleges, universities, community centres and to service users and families. Lay summaries will be made available on the project website and shared via social media sites, i.e. Facebook page, Instagram and twitter to make it available to the wider public. Press releases will be made relating to key project findings. Members of the research team may give interviews relating to the study to the media outlets. Presentations about the research and key findings will also be undertaken with stakeholders linked to the project including clinical services, clinicians and local communities. Furthermore, the overall dissemination will take place after having an adequate dissemination plan which include journal publications.

## Discussion

The protocol describes the process to assess the feasibility and acceptability of MICBT and MBRP (CAMIAB) intervention for individuals with SUD. Although these are evidence based interventions, research evidence on efficacy of these interventions for SUD in LMICs like Pakistan is limited. If proven effective, the trial will strengthen the evidence base for treatment of SUD. Based on the work during the trial period, we will establish a robust platform to conduct capacity building in order to facilitate opportunities for research knowledge deployment and knowledge exchange. It will also help shape clinical practice, government policy and research in relation to supporting individuals with SUD.

### Trial status

This trial has been registered on ClinicalTrials.gov NCT04885569. The trial is not recruiting participants yet.

## Supplementary Information

**Additional file 1.**

## Data Availability

Not applicable.

## Abbreviations

CBT: Cognitive behavioral therapy
MICBT: Cognitive Behavioral Therapy based Motivational Interviewing
MBRP: Mindfulness based Relapse Prevention
SUD: Substance use disorder
LMICs: Lower- and middle-income countries
RCT: Randomized controlled trial
UNODC: United Nations Office on Drugs and Crime
WHO: World Health Organisation
NGO: Non-governmental organization
INL: Bureau of International Narcotics and Law Enforcement Affairs
MAP: The Maudsley Addiction Profile
SWEMWS: The Short Warwick-Edinburgh Mental Well-being Scale
CSRI: Client service receipt inventory

## References

[1] UNODC. World Drug Report 2021. Available from: https://reliefweb.int/attachments/6e655f8c-61e6-3de9-8481-a5097460d891/WDR21_Booklet_1.pdf.

[2] UNODC. World Drug Report 2020. United Nations publication. 2020. Available from https://wdr.unodc.org/wdr2020/field/WDR20_BOOKLET_1.pdf.

[3] UNODC. Drug use in Pakistan 2013: https://www.unodc.org/documents/pakistan/Survey_Report_Final_2013.pdf (last accessed 21 Mar 2022).

[4] Kayani A, King M, Watson B, Karim S. The criminal justice system of Pakistan: deterrent impacts for drug and alcohol use among road drivers. In: Thue L, editor. Proceedings of the 22nd International Council on Alcohol, Drugs and Traffic Safety Conference. Canada: International Council on Alcohol, Drugs and Traffic Safety (ICADTS); 2019. p. 184–8.

[5] MENAHRA. ASSESSMENT OF SITUATION AND RESPONSE OF DRUG USE AND ITS HARMS IN THE MIDDLE EAST AND NORTH AFRIC: http://www.menahra.org/images/pdf/Situation_Assessment_2021_-_Web.pdf (last accessed 21 Mar 2022).

[6] Batool S, Manzoor I, Hassnain S, Bajwa A, Abbas M, Mahmood M, Sohail H (2017). Pattern of addiction and its relapse among habitual drug abusers in Lahore, Pakistan. East Mediterr Health J..

[7] Bureau of International Narcotics and Law Enforcement Affairs. International Narcotics Control Strategy Report: Drug and chemical control 2016: https://2009-2017.state.gov/documents/organization/253655.pdf (last accessed 21 Mar 2022).

[8] Jhanjee S (2014). Evidence based psychosocial interventions in substance use. Indian J Psychol Med.

[9] Zilverstand A, Parvaz MA, Moeller SJ, Goldstein RZ (2016). Cognitive interventions for addiction medicine: understanding the underlying neurobiological mechanisms. Prog Brain Res.

[10] Zamboni L, Centoni F, Fusina F, Mantovani E, Rubino F, Lugoboni F, Federico A (2021). The effectiveness of cognitive behavioral therapy techniques for the treatment of substance use disorders: a narrative review of evidence. J Nerv Ment Dis.

[11] Morin JF, Harris M, Conrod PJ. A review of CBT treatments for substance use disorders. Oxford Handbooks Online. 10.1093/oxfordhb/9780199935291.013.57

[12] Miller WR (1983). Motivational interviewing with problem drinkers. Behav Cogn Psychother.

[13] Kabat-Zinn J (2003). Mindfulness-based stress reduction (MBSR). Constructivism Hum Sci.

[14] Brown S (2010). Mindfulness-Based Relapse Prevention for Addictive Behaviors: a clinician's guide, by Sarah Bowen, Neha Chawla, and G. Alan Marlatt.

[15] Felver JC, Hayes SC, Levin ME, editors. Mindfulness & acceptance for addictive behaviors: applying contextual CBT to substance abuse & behavioral addictions. Oakland: New Harbinger Publications; 2012. p. 336.

[16] Harrison R, Benton T, Everson-Stewart S, Weinstein P (2007). Effect of motivational interviewing on rates of early childhood caries: a randomized trial. Pediatr Dent.

[17] Hogue A, Henderson CE, Becker SJ, Knight DK (2018). Evidence base on outpatient behavioral treatments for adolescent substance use, 2014–2017: outcomes, treatment delivery, and promising horizons. J Clin Child Adolesc Psychol.

[18] Kohler S, Hofmann A (2015). Can motivational interviewing in emergency care reduce alcohol consumption in young people? A systematic review and meta-analysis. Alcohol Alcohol.

[19] Lenz AS, Rosenbaum L, Sheperis D (2016). Meta-analysis of randomized controlled trials of motivational enhancement therapy for reducing substance use. J Addict Offender Counseling..

[20] Hettema J, Steele J, Miller WR (2005). Motivational interviewing. Annu Rev Clin Psychol.

[21] Marlatt GA, Donovan DM, editors. Relapse prevention: maintenance strategies in the treatment of addictive behaviors. New York: Guilford Press; 2005.

[22] Bowen S, Chawla N, Collins SE, Witkiewitz K, Hsu S, Grow J, Clifasefi S, Garner M, Douglass A, Larimer ME, Marlatt A (2009). Mindfulness-based relapse prevention for substance use disorders: a pilot efficacy trial. Substance abuse.

[23] Bowen S, Chawla N, Witkiewitz K. Mindfulness-based relapse prevention for addictive behaviors. In: Baer RA, editor. Mindfulness-based treatment approaches Clinician's guide to evidence base and applications: Elsevier Academic Press; 2014. p. 141–57. 10.1016/B978-0-12-416031-6.00007-4.

[24] Korecki JR, Schwebel FJ, Votaw VR, Witkiewitz K (2020). Mindfulness-based programs for substance use disorders: a systematic review of manualized treatments. Subst Abuse Treat Prev Policy.

[25] Tolin DF (2010). Is cognitive–behavioral therapy more effective than other therapies?: a meta-analytic review. Clin Psychol Rev.

[26] Barrowclough C, Haddock G, Wykes T, Beardmore R, Conrod P, Craig T, Davies L, Dunn G, Eisner E, Lewis S, Moring J (2010). Integrated motivational interviewing and cognitive behavioural therapy for people with psychosis and comorbid substance misuse: randomised controlled trial. BMJ.

[27] Naar S, Safren SA (2017). Motivational interviewing and CBT: combining strategies for maximum effectiveness.

[28] Bowen S, Chawla N, Marlatt AG (2011). Mindfulness-based relapse prevention for addictive behaviors: A clinicians’ guide.

[29] Sancho M, De Gracia M, Rodríguez RC, Mallorquí-Bagué N, Sánchez-González J, Trujols J, Sánchez I, Jiménez-Murcia S, Menchón JM (2018). Mindfulness-based interventions for the treatment of substance and behavioral addictions: a systematic review. Front Psych.

[30] Braun V, Clarke V (2006). Using thematic analysis in psychology. Qual Res Psychol.

[31] Rathod S, Gega L, Degnan A, Pikard J, Khan T, Husain N, Munshi T, Naeem F (2018). The current status of culturally adapted mental health interventions: a practice-focused review of meta-analyses. Neuropsychiatr Dis Treat.

[32] Naeem F, Saeed S, Irfan M, Kiran T, Mehmood N, Gul M, Munshi T, Ahmad S, Kazmi A, Husain N, Farooq S (2015). Brief culturally adapted CBT for psychosis (CaCBTp): a randomized controlled trial from a low income country. Schizophr Res.

[33] Naeem F, Gul M, Irfan M, Munshi T, Asif A, Rashid S, Khan MN, Ghani S, Malik A, Aslam M, Farooq S (2015). Brief culturally adapted CBT (CaCBT) for depression: a randomized controlled trial from Pakistan. J Affect Disord.

[34] Husain N, Afsar S, Ara J, Fayyaz H, ur Rahman R, Tomenson B, Hamirani M, Chaudhry N, Fatima B, Husain M, Naeem F (2014). Brief psychological intervention after self-harm: randomised controlled trial from Pakistan. Br J Psychiatr.

[35] Naeem F, Habib N, Gul M, Khalid M, Saeed S, Farooq S, Munshi T, Gobbi M, Husain N, Ayub M, Kingdon D (2016). A qualitative study to explore patients’, carers’ and health professionals’ views to culturally adapt CBT for psychosis (CBTp) in Pakistan. Behav Cogn Psychother.

[36] Lovell K, Wearden A, Bradshaw T, Tomenson B, Pedley R, Davies LM, Husain N, Woodham A, Escott D, Swarbrick CM, Femi-Ajao O (2014). An exploratory randomized controlled study of a healthy living intervention in early intervention services for psychosis: the INTERvention to encourage ACTivity, improve diet, and reduce weight gain (INTERACT) study. J Clin Psychiatry.

[37] Husain MI, Chaudhry IB, Khoso AB, Wan MW, Kiran T, Shiri T, Chaudhry N, Mehmood N, Jafri SF, Naeem F, Husain N (2021). A group parenting intervention for depressed fathers (LTP+ Dads): a feasibility study from Pakistan. Children.

[38] Husain N, Zulqernain F, Carter LA, Chaudhry IB, Fatima B, Kiran T, Chaudhry N, Naeem S, Jafri F, Lunat F, Haq SU (2017). Treatment of maternal depression in urban slums of Karachi, Pakistan: a randomized controlled trial (RCT) of an integrated maternal psychological and early child development intervention. Asian J Psychiatr.

[39] Husain N, Kiran T, Fatima B, Chaudhry IB, Husain M, Shah S, Bassett P, Cohen N, Jafri F, Naeem S, Zadeh Z (2021). An integrated parenting intervention for maternal depression and child development in a low-resource setting: cluster randomized controlled trial. Depress Anxiety.

[40] Moyers TB, Houck J (2011). Combining motivational interviewing with cognitive-behavioral treatments for substance abuse: Lessons from the COMBINE research project. Cogn Behav Pract.

[41] Garland EL, Howard MO (2018). Mindfulness-based treatment of addiction: current state of the field and envisioning the next wave of research. Addict Sci Clin Pract.

[42] Tennant R, Hiller L, Fishwick R, Platt S, Joseph S, Weich S, Parkinson J, Secker J, Stewart-Brown S (2007). The Warwick-Edinburgh mental well-being scale (WEMWBS): development and UK validation. Health Qual Life Outcomes.

[43] Brooks R (2017). EQ-5D and the EuroQol group: past, present and future. Appl Health Econ Health Policy.

[44] Chisholm D, Knapp MR, Knudsen HC, Amaddeo F, Gaite LU, Van Wijngaarden BO, EPSILON Study Group (2000). Client socio-demographic and service receipt inventory–European version: development of an instrument for international research: EPSILON Study 5. Br J Psychiatr.

[45] Sim J, Lewis M (2012). The size of a pilot study for a clinical trial should be calculated in relation to considerations of precision and efficiency. J Clin Epidemiol.

[46] Efird J (2011). Blocked randomization with randomly selected block sizes. Int J Environ Res Public Health.

[47] Marsden J, Gossop M, Stewart D, Best D, Farrell M, Lehmann P, Edwards C, Strang J (1998). The Maudsley Addiction Profile (MAP): a brief instrument for assessing treatment outcome. Addiction.

[48] Scott CK (2004). A replicable model for achieving over 90% follow-up rates in longitudinal studies of substance abusers. Drug Alcohol Depend.

[49] Ward DJ, Furber C, Tierney S, Swallow V (2013). Using F ramework A nalysis in nursing research: a worked example. J Adv Nurs.

[50] Tong A, Sainsbury P, Craig J (2007). Consolidated criteria for reporting qualitative research (COREQ): a 32-item checklist for interviews and focus groups. Int J Qual Health Care.

